# Adverse Childhood Experiences Among 28,047 Norwegian Adults From a General Population

**DOI:** 10.3389/fpubh.2021.711344

**Published:** 2021-07-26

**Authors:** Siri H. Haugland, Anders Dovran, Ane U. Albaek, Børge Sivertsen

**Affiliations:** ^1^Department of Psychosocial Health, University of Agder, Grimstad, Norway; ^2^Stine Sofies Foundation and Stine Sofie Centre, Grimstad, Norway; ^3^Department of Health Promotion, Norwegian Institute of Public Health, Bergen, Norway; ^4^Department of Research and Innovation, Helse Fonna HF, Haugesund, Norway; ^5^Department of Mental Health, Norwegian University of Science and Technology, Trondheim, Norway

**Keywords:** adverse childhood experiences, family conflict, adult survivors of child adverse events, child abuse, socioeconomic factors

## Abstract

**Aim:** The purpose of this study was to estimate the prevalence of adverse childhood experiences (ACEs) among Norwegian adults from a general population and to identify potential associations with demographic and socioeconomic characteristics.

**Methods:** A randomly drawn sample (*N* = 61,611) from the public registry of inhabitants was invited to participate in the Norwegian Counties Public Health Survey. The present study was based on online responses from 28,047 adults ≥18 years (mean age: 46.9 years, SD = 16.03). Log-link binomial regression analyses were performed to examine associations between four measures of ACEs (family conflict, lack of adult support, bad memories, and difficult childhood) and demographic (age, gender, civil status, parental divorce) and socioeconomic characteristics (education level, perceived financial situation, and welfare benefits).

**Results:** Single individuals and those with parents that divorced during childhood were at elevated risk of all four ACEs. The risk varied to some degree between the sexes. The prevalence of ACEs declined with increasing age. We found a consistent social gradient that corresponded to the frequency of ACEs for all three socioeconomic characteristics investigated. The risks were highest for those in the lowest socioeconomic levels (RR: 1.53, 95% CI: 1.32–1.78 to RR: 4.95, CI: 4.27–5.74).

**Conclusions:** Public health strategies should direct more attention to the interplay between ACEs and socioeconomic factors. Welfare services should be sensitive to ACEs among their service recipients.

## Introduction

Adverse childhood experiences (ACEs) are stressful and potentially traumatic events experienced by children before the age of 18 years. ACEs are relatively common (1, 2): a recent systematic review and meta-analysis found pooled prevalence of 23.5% Europeans that reported at least one ACE and 18.7% that reported two or more ACEs (3). The term “ACE” originated in the Adverse Childhood Experiences study, conducted in 1998. They grouped ACEs into three domains: abuse, neglect, and household dysfunction (4). Numerous studies have explored the consequences of ACEs, although, the concept has been defined differently among different studies (5). Typically, studies map the occurrence, number, and frequency of various types of adverse experiences (3). Other studies focused on the relevance of how participants rated the impact of their experiences (6). However, irrespective of how ACEs have been defined, the association between ACEs and reduced adult health and well-being has been confirmed repeatedly (4–8). Although, a large body of research has documented the connection between early adversities and adult health problems, fewer studies have explored factors that could influence and interact with this connection. One example is the complex relationship between ACEs and socioeconomic factors. ACEs appear to be highly socially patterned and individuals with low socioeconomic statuses report more ACEs (9). However, due to their occurrence early in life, it is likely that ACEs also impact socioeconomic outcomes in adulthood, such as educational attainment, employment, and income (10, 11). Although, Norway is considered an affluent country, we nevertheless display a social gradient in health and life expectancy (12, 13). The number of children living in low income families (below the poverty threshold) in Norway has tripled over the last two decades, and more people receive disability benefits here compared to other countries that are members of the Organization for Economic Co-operation and Development (14). In addition, Norway has regional differences in education, disability, and health-related measures. The current study was situated in a region characterized by low employment rates, a high distribution of work assessment allowance (unemployment benefit), and a large proportion of people that receive disability benefits. Moreover, among young adults (15–29 years) in this area, the frequency of seeking help for mental health problems was higher than the national average (14). Few studies have investigated the prevalence of ACEs and their association with socioeconomic and demographic factors in a Nordic context and in a general population of adults. However, this information is important to improve our understanding of the inequalities and determinants of health in Western societies. Epidemiological studies that identify high-risk groups are essential in developing policy and service-delivery systems directed toward reducing the negative consequences of ACEs (15).

The purpose of this study was to estimate the prevalence of adverse childhood experiences (ACEs) among Norwegian adults from a general population and to identify potential associations with demographic and socioeconomic characteristics.

## Methods

### Study Design and Setting

A total of 61,611 inhabitants that resided in Agder county (Southern Norway), aged 18 or older, were invited to participate in an online questionnaire (Norwegian Counties Public Health Survey). The questions were related to health, well-being, childhood, living conditions, local environments, accidents, and injuries. All data were collected electronically through a web-based platform. Participants were selected randomly from the Norwegian Population Registry of inhabitants in Southern Norway; e-mails or telephone numbers were obtained from the contact registry from the Norwegian Agency for Public Management and eGovernment.

### Instruments

#### Adverse Childhood Experiences

ACEs were assessed with these four questionnaire items:

1. *Did you experience a lot of arguing, turmoil, conflicts, or difficult communication in your childhood home?*
2. *Growing up, did you have a trusted adult from whom you could get support?*
3. *Do you struggle with bad memories from your childhood, due to loss, betrayal, neglect, violence, ill-treatment, or abuse?*
4. *When you think about your childhood/upbringing, how would you describe it?*

Response options for items 1–3 were: “not at all,” “to a very small degree,” “to a small degree,” “to a large degree,” and “to a very large degree.” The last three categories in item 1 were coded as a “dysfunctional family environment”; the first two categories in item 2 were coded as a “lack of trusted adult during childhood”; and the last three categories in item 3 were coded as a “struggle with bad memories.” Item 4 included the following response categories “very good,” “good,” “moderate,” “difficult,” and “very difficult.” The last two categories were coded as “perceived childhood as difficult.” Finally, one item assessed the participant's experience with parental divorce, with the response options “no,” “yes, before I was 7 years old,” and “yes, when I was between 7 and 18 years old.”

These four ACE items were originally developed for a large Norwegian public health study (the HUNT study). Items 1–3 first appeared in the fourth wave of the study (HUNT4), and item 4 was included in both the third and fourth waves (HUNT3 and HUNT4). Before that, the ACE items were included in a pilot-testing of the HUNT4 questionnaire in the municipality of Selbu, where, 31 participants provided written comments to the questions. In addition, six participants were interviewed in detail by telephone. Of particular interest, the pilot study tested the comprehensibility of the questions and whether participants found them uncomfortable or invasive. No negative comments regarding the ACE items were received.

In a previous study, items 1, 3, and 4 were validated together in a short, Difficult Childhood questionnaire (DCQ). The discriminant and convergent validities of the tool were confirmed (16) in the same population that we analyzed in the current study.

#### Demographic Factors

Participant age and sex were obtained from the National Population Registry. Age was divided into 6 age groups (18–29, 30–39, 40–49, 50–59, 60–69, and 70+ years). All participants were also asked about their relationship status (coded as “single” vs. “married/partner” (including “girlfriend/boyfriend”).

#### Socioeconomic Variables

Three variables measured different aspects of socioeconomic status (SES). The educational level was collected by asking participants about the highest level of education completed. The response categories were: “Low secondary/secondary modern/folk high school up to 10 years,” “Vocational training/middle school/upper secondary/junior college- minimum 3 years,” “University college/university <4 years,” and “University college/university 4 years or more.” In the current study, these categories were renamed “low,” “medium,” “high,” and “highest” educational level, respectively. Economic capability was assessed with the following question: “How easy or difficult is it for you to make ends meet with your current income?” Response options ranged from 1 (very difficult) to 6 (very easy). For the purposes of the current study, these responses were revised to include three categories: “poor,” “medium,” “good.” Participants were also asked whether their employment status included receiving disability pension/work assessment allowance or social assistance benefits. These categories were collapsed into two: “receiving welfare benefits” or “not receiving welfare benefits.” As this last variable allowed the participants to tick off several answers, missing is not possible to estimate for this particular variable.

### Ethics

This study was approved by the Norwegian Data Inspectorate and the Regional Committee for Medical and Health Research Ethics of South-East Norway (file number 162353/REK South-East-C), whose directives are based on the Helsinki Declaration. Written electronic consent was provided by all subjects included in the study. All data were stored and processed in compliance with The General Data Protection Regulation.

### Statistical Analysis

All analyses were performed with IBM SPSS version 26 (SPSS Inc., Chicago, IL USA) for Windows. The overall distribution of ACEs, relative to demographic and socioeconomic factors were performed in cross-tables. Log-link binomial regression analyses were performed to examine associations between ACEs and demographic and socioeconomic factors. Rather than the more commonly used logistic regression model to obtain an odds ratio (OR), we used log-link binomial regressions, to obtain risk ratios (RR), and 95% confidence intervals (95% CIs). All analyses were conducted separately for males and females. We also tested for interactions between sex and the other demographic and socioeconomic factors by entering the product of these variables in separate blocks. Missing data were handled with a listwise deletion method.

## Results

Of the 61,611 individuals invited, 28,047 completed the questionnaire, which yielded a response rate of 45.5%. The sample had a mean age of 46.9 years (SD = 16.03) and consisted of 13,122 (46.8%) males and 14,925 females (53.2%).

**Figure 1:**
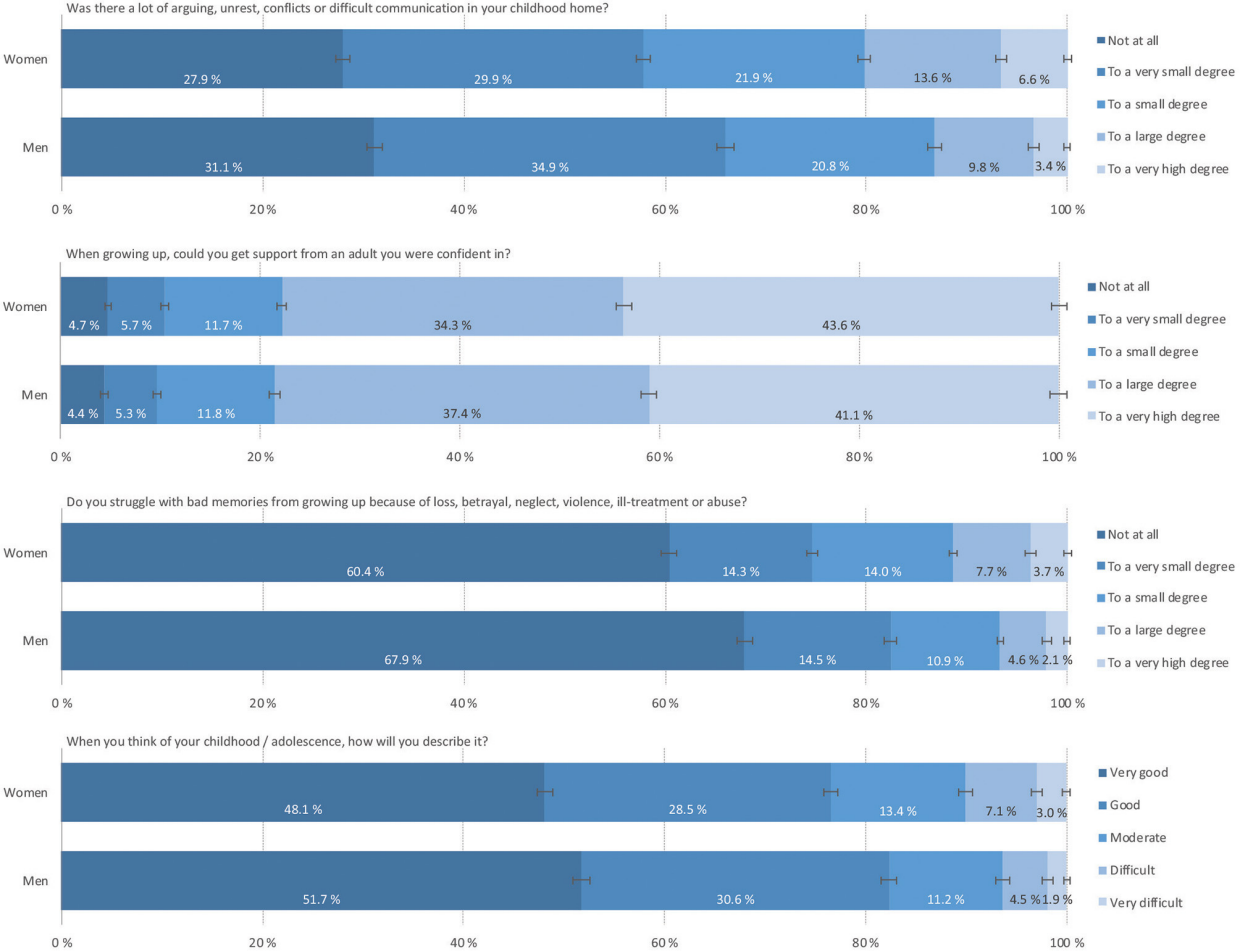
The prevalence of ACEs among Norwegian adults from a general population, grouped by participant sex. Data retrieved from the Norwegian Counties Public Health Survey conducted in Agder, 2019.

Figure 1 shows the prevalence of the different ACEs. More females (20.2%) than males (13.2%) reported frequent family conflicts. In contrast, the sexes were less different in the support perceived; approximately 10% reported a lack of support (small to very small degrees of support). Struggling with bad memories from childhood was reported by 11.4% of females and 6.7% of males. Similarly, 10.1% of females and 6.4% of males characterized their childhood as difficult or very difficult.

A comparison between the different age groups showed a declining trend, where older age groups reported less ACEs than younger age groups (data not shown). For all the ACE-related variables, females reported higher frequencies than males.

**Figure 2:**
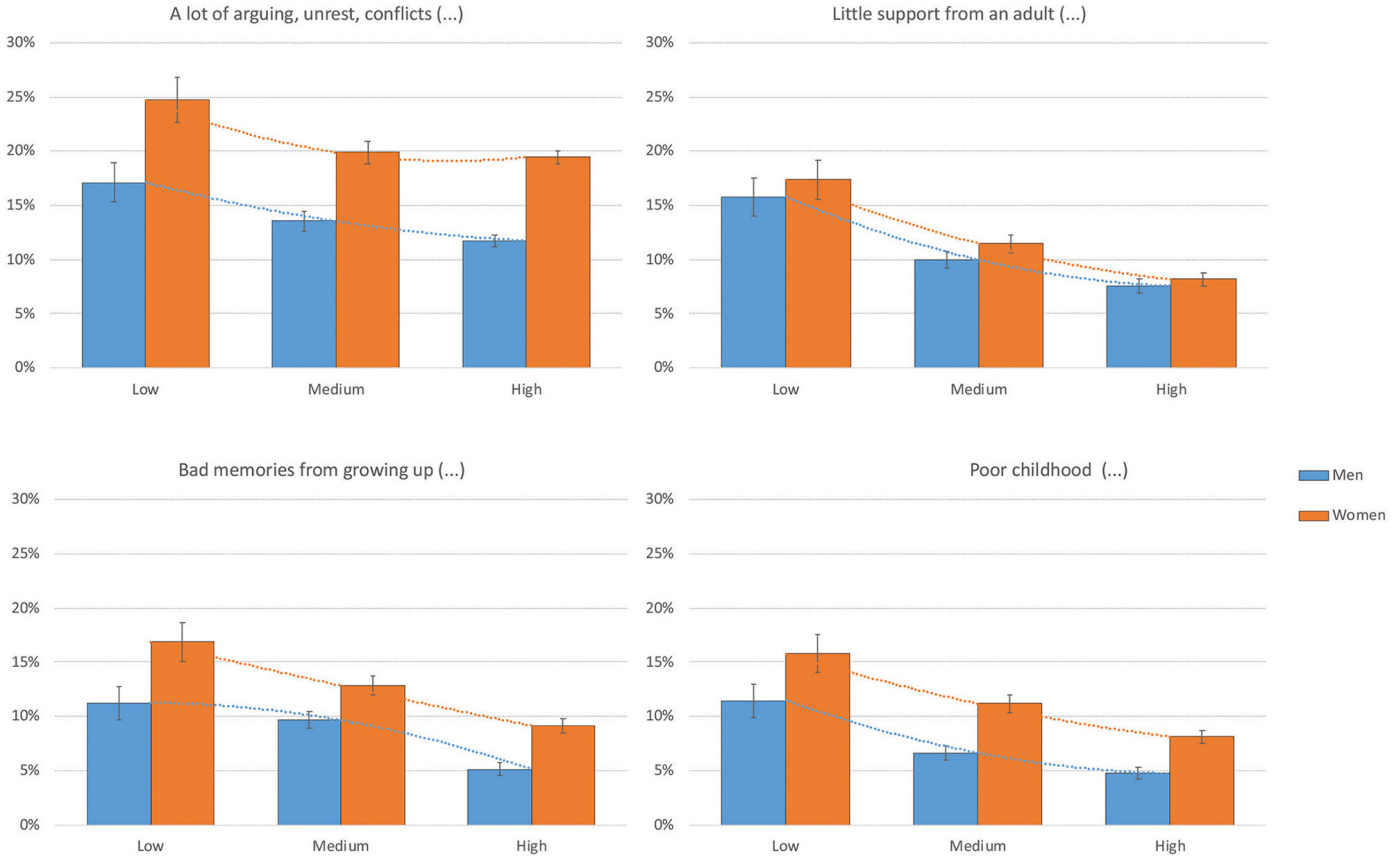
The prevalence of ACEs among Norwegian adults from a general population, grouped by education. Data retrieved from the Norwegian Counties Public Health Survey conducted in Agder, 2019.

Table 1 shows the percentages of individuals that reported the four ACEs among different demographic and socioeconomic groups. Within the socioeconomic groups, the highest proportions of participants that reported ACEs were in the lower socioeconomic groups (low education levels, poor economic capability, and recipients of welfare benefits) and came from a background with parental divorce. Figure 2 visualize the social gradient in the prevalence of ACE by education.

**Table 1:** Demographic and socioeconomic characteristics of adults with adverse childhood experiences; data from a Norwegian Counties Public Health Survey conducted in Agder, 2019 (*N* = 28,047).

**Characteristic**	**Dysfunctional family environment** ***n* (%)**	**Lack of trusted adult during childhood** ***n* (%)**	**Struggle with bad memories** ***n* (%)**	**Perceives childhood as difficult** ***n* (%)**
**Sex**				
Male	1,725 (13.2)	2,802 (21.5)	873 (6.7)	841 (6.4)
Female	3,008 (20.2)	3,290 (22.1)	1,696 (11.4)	1,505 (10.1)
**Age group**				
18–29 years	1,077 (20.7)	997 (19.1)	641 (12.3)	526 (10.1)
30–39 years	970 (21.6)	1,040 (23.2)	548 (12.2)	514 (11.4)
40–49 years	1,062 (19.2)	1,261 (22.8)	534 (9.6)	526 (9.5)
50–59 years	944 (16.9)	1,308 (23.5)	494 (8.8)	474 (8.5)
60–69 years	505 (10.7	1,000 (21.2)	256 (5.4)	225 (4.8)
70 years +	175 (7.3)	486 (20.2)	96 (4.0)	81 (3.3)
**Parents divorced during childhood**				
Yes, age <7 years	1,038 (44.0)	889 (37.7)	623 (26.4)	599 (25.3)
Yes, age 7–18 years	1,201 (43.4)	990 (35.8)	559 (20.2)	562 (20.3)
No	2,476 (10.9)	4,191 (18.4)	1,381 (6.1)	1,175 (5.2)
**Marital status**				
Single	1,291 (21.3)	1,626 (26.8)	807 (13.3)	760 (12.5)
Married/partner	3,432 (15.7)	4,451 (20.4)	1,757 (8.0)	1,582 (7.2)
**Educational level**				
Low	696 (20.9)	1,001 (30.3)	468 (14.1)	454 (13.6)
Medium	1,847 (16.7)	2,528 (22.9)	1,088 (9.8)	985 (8.9)
High	1,067 (16.4)	1,317 (20.3)	524 (8.1)	464 (7.1)
Highest	1,105 (15.8)	1,221 (17.5)	476 (6.8)	433 (6.2)
**Financial difficulties**				
Poor	768 (32.6)	923 (39.1)	610 (25.8)	548 (23.2)
Medium	812 (25.6)	962 (30.3)	493 (15.5)	432 (13.6)
No difficulty	2,978 (14.1)	3,923 (18.6)	1,360 (6.4)	1,267 (6.0)
**Welfare Benefits**				
Yes	1,006 (31.4)	1,180 (37.0)	819 (25.6)	702 (21.9)

As detailed in Table 2, participants that reported that their parents had divorced during childhood had an elevated overall risk for all four ACEs, with RRs ranging from 1.85 to 5.62. We found that this association significantly interacted with sex; indeed, males generally showed stronger associations than females between having divorced parents and experiencing three of the four ACE outcomes: a dysfunctional family environment, struggling with bad memories, and perceiving childhood as difficult. The risk of ACEs declined with age; it was lowest among the oldest participants. We also found that single individuals (without partner) showed elevated risks for all four ACEs, compared to individuals with a partner (RRs ranged from 1.26 to 1.92). We also found that males generally had stronger associations than females between a single marital status and a dysfunctional family environment and perceiving childhood as difficult (Table 2).

**Table 2:** Risk of adverse childhood experiences and demographic and socioeconomic characteristics, among Norwegian adults from a general population; data from Norwegian Counties Public Health Survey conducted in Agder 2019 (*N* = 28,047).

**Characteristic**	**Dysfunctional family environment**				**Lack of trusted adult during childhood**				**Struggle with bad memories**				**Perceives childhood as difficult**			
	**Males**		**Females**		**Males**		**Females**		**Males**		**Females**		**Males**		**Females**	
	**RR**	**95% CI**	**RR**	**95% CI**	**RR**	**95% CI**	**RR**	**95% CI**	**RR**	**95% CI**	**RR**	**95% CI**	**RR**	**95% CI**	**RR**	**95% CI**
**Age group**	Sex int. Wald (df) = 0.982(5), *p =* 0.964				Sex int. Wald (df) = 8.331(5), *p =* 0.139				Sex int. Wald (df) = 6.676(5), *p =* 0.246				Sex int. Wald (df) = 7.014(5), *p =* 0.220			
18–29 years	1.00	–	1.00	–	1.00	–	1.00	–	1.00	–	1.00	–	1.00	–	1.00	–
30–39 years	1.04	0.907–1.19	1.06	0.967–1.16	1.18	1.05–1.34	1.23	1.11–1.36	1.14	0.944–1.38	0.942	0.828–1.07	1.24	1.01–1.51	1.09	0.950–1.26
40–49 years	0.918	0.804–0.1.08	0.963	0.879–1.05	1.15	1.02–1.29	1.23	1.18–1.36	0.791	0.648–0.964	0.814	0.716–0.926	0.877	0.716–1.07	1.01	0.879–1.16
50–59 years	0.824	0.72–0.943	0.849	0.77–93	1.23	1.10–1.38	1.22	1.11–1.34	0.783	0.643–0.953	0.725	0.633–0.829	0.848	0.693–1.03	0.873	0.754–1.01
60–69 years	0.527	0.449–0.618	0.547	0.483–0.620	1.17	1.04–1.32	1.04	0.939–1.16	0.473	0.374–0.598	0.462	0.388–0.550	0.468	0.365–0.599	0.509	0.421–0.617
70 years +	0.346	0.274–0.437	0.399	0.326–0.488	1.11	0.973–1.27	0.999	0.865–1.15	0.302	0.214–0.427	0.395	0.304–0.513	0.295	0.204–0.426	0.410	0.306–0.550
**Parents divorced during childhood**	Sex int. Wald (df) = 39.833(2), *p =* 0.000				Sex int. Wald (df) = 1.042(2), *p =* 0.594				Sex int. Wald (df) = 6.255(2), *p =* 0.044				Sex int. Wald (df) = 6.390(2), *p =* 0.041			
Yes, age <7 years	4.77	4.14–5.05	3.58	3.34–3.83	1.85	1.63–2.09	2.01	1.81–2.23	3.49	2.97–4.11	3.11	2.80–3.47	4.10	3.49–4.82	3.71	3.24–4.24
Yes, age 7–18 years	5.05	4.58–5.76	3.45	3.21–3.72	2.04	1.79–2.31	2.04	1.83–2.29	4.94	4.26–5.72	3.94	3.55–4.36	5.62	4.84–6.51	4.42	3.96–4.94
No	1.00	–	1.00	–	1.00	–	1.00	–	1.00	–	1.00	–	1.00	–	1.00	–
**Marital status**	Sex int. Wald (df) = 4.77(1), *p =* 0.029				Sex int. Wald (df) = 0.04(1), *p =* 0.307				Sex int. Wald (df) = 0.007(1), *p =* 0.931				Sex int. Wald (df) = 4.01(1), *p =* 0.045			
Single	1.47	1.31–1.65	1.25	1.14–1.37	1.26	1.14–1.39	1.35	1.24–1.47	1.83	1.58–2.13	1.51	1.35–1.69	1.92	1.65–2.23	1.58	1.41–1.77
Married/partner	1.00	–	1.00	–	1.00	–	1.00	–	1.00	–	1.00	–	1.00	–	1.00	–
**Educational level**	Sex int. Wald (df) = 5.741(3), *p =* 0.125				Sex int. Wald (df) = 0.689(3), *p =* 0.876				Sex int. Wald (df) = 39.833(3), *p =* 0.225				Sex int. Wald (df) = 7.406(3), *p =* 0.006			
Low	1.53	0.1.32–1.78	1.28	1.16–1.43	1.68	1.51–1.88	1.71	1.60–1.95	2.39	1.93–2.96	2.02	1.75–2.35	2.42	1.96–3.00	2.18	1.86–2.54
Medium	1.21	1.07–1.37	1.03	0.956–1.127	1.29	1.18–1.42	1.32	1.21–1.43	1.46	1.21–1.77	1.54	1.36–1.74	1.40	1.16–1.70	1.54	1.35–1.76
High	1.11	966–1.28	1.02	0.939–1.128	1.12	1.01–1.25	1.18	1.08–1.30	1.18	0.949–1.47	1.21	1.05–1.39	0.995	0.79–1.25	1.26	1.08–1.46
Highest	1.00	–	1.00	–	1.00	–	1.00	–	1.00	–	1.00	–	1.00	–	1.00	–
**Economic capabilities**	Sex int. Wald (df) = 2.146(2), *p =* 0.342				Sex int. Wald (df) = 0.590(2), *p =* 0.745				Sex int. Wald (df) = 4.36(2), *p =* 0.000				Sex int. Wald (df) = 4.179(2), *p =* 0.124			
Poor	2.40	2.13–2.70	2.18	2.01–2.37	2.07	1.89–2.25	2.12	1.96–2.29	4.95	4.27–5.74	3.46	3.11–3.84	4.29	3.68–5.01	3.53	3.15–3.94
Medium	1.87	1.66–2.10	1.74	1.60–1.89	1.58	1.44–1.73	1.66	1.53–1.79	2.70	2.28–3.20	2.21	1.97–2.48	2.29	1.92–2.74	2.19	1.93–2.
Good	1.00	–	1.00	–	1.00	–	1.00	–	1.00	–	1.00	–	1.00	–	1.00	–
**Welfare benefits**	Sex int. Wald (df) = 5.144(1), *p =* 0.023				Sex int. Wald (df) = 0.234(1), *p =* 0.622				Sex int. Wald (df) = 6.297(1), *p =* 0.012				Sex int. Wald (df) = 4.244(1), *p =* 0.039			
Yes	2.45	1.90–2.54	1.90	1.72–2.09	1.91	1.69–2.15	1.84	1.67–2.01	4.08	3.47–4.78	3.18	2.84–3.55	3.65	3.09–4.31	2.95	2.62–3.32
No	1.00	–	1.00	–	1.00	–	1.00	–	1.00	–	1.00	–	1.00	–	1.00	–

The risk of ACEs was highest in disadvantaged subgroups (i.e., those with a low education level, poor economic capability, or recipients of welfare benefits), with RRs ranging from 1.28 (95% CI: 1.16–1.43) to 4.95 (95% CI: 4.27–5.74). Among participants with poor economic capability and participants that received welfare benefits, males were at significantly higher risk than females of struggling with bad memories. Similarly, among participants with low education and those that received welfare benefits, males were at significantly higher risk than females of perceiving childhood as difficult (Table 2).

## Discussion

Overall, our results showed that that the prevalence of ACEs (family conflict, lack of adult support, struggling with bad memories, and difficult childhood) in a large Norwegian adult sample drawn from the general population varied across demographic variables (i.e., age, gender, marital status, and a background of divorced parents). We also showed that exposure to childhood adversities was associated with low socioeconomic status in adulthood, including variables like low education levels, perceived financial difficulties, and receiving welfare benefits.

In our sample, the proportions of males and females that reported ACEs varied with age, where few of the oldest participants reported ACEs. Although, this result was consistent with results from previous studies (17), it may be somewhat surprising, because one might have expected that childhood conditions would have been worse among the oldest individuals. There are several possible explanations for this age-related decline in ACEs. First, the questions were retrospective, and therefore, they were susceptible to recall bias (18); this bias might have been more pronounced among the oldest participants. Second, studies have shown that ACEs were strongly related to multimorbidity (18) and premature mortality (17). This association may have introduced a selection bias, where the oldest individuals with high levels of ACEs might have been underrepresented. Furthermore, there may be differences between age cohorts in their understanding of what qualifies as a difficult childhood and their expectations of how childhood should be. Although, these are plausible explanations for our findings, another Norwegian study (18) evaluated one of our ACE items (a difficult childhood) and did not find any significant differences between age groups in the levels of self-reported childhood difficulties.

Single participants had a modestly increased risk of ACEs compared to those with a partner. Childhood adversities, such as family conflicts, might influence relationship aspects. For example, a study by Roth et al. (19) found that general confidence in sustainable romantic relationships was lower among individuals that were exposed to overt parental conflict during childhood.

Not surprisingly, individuals that experienced a parental divorce during childhood had a higher risk of ACEs than those with parents that did not divorce. Furthermore, adults that experienced a parental divorce during childhood were more prone to struggling with bad memories from their childhood due to loss, betrayal, neglect, violence, ill treatment, or abuse. This finding was consistent with other studies that revealed long-term associations between parental divorce and a wide range of different mental health problems (20).

Overall, our findings with socioeconomic measures revealed a strong, consistent social gradient that corresponded to the risks of all four ACEs. Among participants that reported financial difficulties, the risk of also struggling with bad memories from childhood was particularly increased among males, but the overall association was strong for both sexes. Similarly, participants with financial difficulties were at high risk of characterizing their childhood as difficult, although, this association was strongest among males. Among participants that received welfare benefits, the pattern was similar, with a pronounced increase in the risk of ACEs. The relationship between education level and ACEs also revealed a gradient; indeed, compared to participants with a high education level, participants with medium and low education levels were at increased risks of all ACEs. These associations were strongest among males with a low education level that characterized their childhood as difficult. The social gradient may have a multifactorial origin, although, it was not possible to investigate this hypothesis in the current study. Other studies have revealed that the prevalence of ACEs was highly socially patterned in childhood, which suggested that a low SES in childhood could be a determinant for ACEs (21). Therefore, a low SES in adulthood might represent a continuance of a low family SES during childhood. However, a previous prospective study (22) found that ACEs were associated with low educational attainment, even after adjusting for family socioeconomic factors.

### Strengths and Limitations

A major strength of this study was the large sample drawn randomly from a general population, including participants that spanned a large age range. Many previous ACE-related studies focused on mapping the types and frequencies of ACEs. In contrast, the present study invited participants to report self-perceptions of the consequences and severity of ACEs, which may be more relevant in assessing experiences that cause detrimental effects on adult perceptions of quality of life (6, 16).

Although, the study is performed within a Norwegian context, we expect findings to be generalizable to other countries as well. Studies performed in other countries have also revealed a social gradient related to ACEs (9, 11). Moreover, as Norway has a well-functioning welfare system compared to many other countries, the social gradient may even be stronger in contexts outside Norway. The Norwegian health services system is mostly government operated and is freely available to all citizens for a minor self-share fee (maximum 240 € per year including medical supplies). Our social services include graded benefits for adults without employment; 1 year sickness benefit [100% of former wage (FW)], 1 year unemployment benefit (62,4% of FW), 3 year work clearance allowance (66% of FW), disability benefit (66% of FW or a minimum amount), and social benefits (cover necessities).

The limitations of this study include its retrospective design. Thus, recall bias might have increased the risk of measurement error (23). However, a comparison between prospective vs. retrospective reports of ACEs did not reveal any bias in our retrospective assessment (24). Another limitation was the cross-sectional study design; caution should be taken regarding potential causalities when interpreting our findings.

### Policy and Practice Implications

Our study indicates that adults who think of their childhood as difficult often experience financial and employment problems. Adding this to our knowledge that adults with ACEs have increased risk for various health problems (5, 25) ACEs impose large human and economic costs on society. The relation between childhood adversity and lifelong well-being warrants fresh thinking on how to promote health and prevent structural inequities. As of now, the majority of societies' resources are allocated to adult health care and adult social services. We suggest that a redirection of resources toward prevention of ACEs, as well as protection and care for children experiencing adversity, will reduce overall human and economic costs. Moreover, interventions focused toward restoring inequities in SES to break intergenerational transmission of low SES and ACEs need to be explored.

## Conclusion

This study showed a varied distribution of ACEs across demographic variables. In addition, a strong, consistent social gradient was revealed, which point to the necessity of increasing our awareness of the potential role that ACEs play in disturbing the life opportunities of children. This awareness should encourage political discourse to increase efforts to disrupt intergenerational patterns, where low SES and ACEs are transferred and upheld within families. The apparent interconnectivity between ACEs and SES calls for a more diverse set of preventive interventions directed toward both SES-related struggles and toward the prevention and treatment of ACEs, for both adults and children.

## Data Availability Statement

The datasets presented in this article are not readily available because the data were provided by the NIPH, with permission. NIPH will make data available in a repository upon application. Requests to access the datasets should be directed to https://helsedata.no/en/.

## Ethics Statement

The studies involving human participants were reviewed and approved by Regional Committee for Medical and Health Research Ethics of South-East-C, Norway (file number 162353). The patients/participants provided their written informed consent to participate in this study.

## Author Contributions

SH, AA, BS, and AD: conceptualization and writing–original draft. BS, AD, and SH: formal analysis and methodology. BS: visualization. All authors contributed to the article and approved the submitted version.

## Conflict of Interest

The authors declare that the research was conducted in the absence of any commercial or financial relationships that could be construed as a potential conflict of interest.

## Publisher's Note

All claims expressed in this article are solely those of the authors and do not necessarily represent those of their affiliated organizations, or those of the publisher, the editors and the reviewers. Any product that may be evaluated in this article, or claim that may be made by its manufacturer, is not guaranteed or endorsed by the publisher.

## References

[1] Merrick MT Ports KA Ford DC Afifi TO Gershoff ET Grogan-Kaylor A. Unpacking the impact of adverse childhood experiences on adult mental health. Child Abuse Negl. (2017) 69:10–9. 10.1016/j.chiabu.2017.03.016 28419887 PMC6007802

[2] WHO. Investing in Children: the European Child Maltreatment Prevention Action Plan 2015–2020. Copenhagen: World Health Organization (2014).

[3] Bellis MA Hughes K Ford K Rodriguez G Sethi D Passmore J. Life course health consequences and associated annual costs of adverse childhood experiences across Europe and North America: a systematic review and meta-analysis. Lancet Public Health. (2019) 4:e517–28. 10.1016/S2468-2667(19)30145-8 31492648 PMC7098477

[4] Felitti VJ Anda RF Nordenberg D Williamson DF Spitz AM Edwards V. Relationship of childhood abuse and household dysfunction to many of the leading causes of death in adults. The Adverse Childhood Experiences (ACE) study. Am J Prev Med. (1998) 14:245–58. 10.1016/S0749-3797(98)00017-8 9635069

[5] Hughes K Bellis MA Hardcastle KA Sethi D Butchart A Mikton C. The effect of multiple adverse childhood experiences on health: a systematic review and meta-analysis. Lancet Public Health. (2017) 2:e356–66. 10.1016/S2468-2667(17)30118-4 29253477

[6] LaNoue M Graeber DA Helitzer DL Fawcett J. The relationship between self-reported adult impact of adverse childhood events and health-related quality of life. J Community Med Health Educ. (2013) 4:267. 10.4172/2161-0711.1000267

[7] Ferrara P Guadagno C Sbordone A Amato M Spina G Perrone G. Child abuse and neglect and its psycho-physical and social consequences: a review of the literature. Curr Pediatr Rev. (2016) 12:301–10. 10.2174/1573396312666160914193357 27634538

[8] Bellis MA Hughes K Leckenby N Hardcastle KA Perkins C Lowey H. Measuring mortality and the burden of adult disease associated with adverse childhood experiences in England: a national survey. J Public Health. (2015) 37:445–54. 10.1093/pubmed/fdu065 25174044 PMC4552010

[9] Metzler M Merrick MT Klevens J Ports KA Ford DC. Adverse childhood experiences and life opportunities: shifting the narrative. Child Youth Serv Rev. (2017) 72:141-9. 10.1016/j.childyouth.2016.10.021 PMC10642285 37961044

[10] Gilbert R Widom CS Browne K Fergusson D Webb E Janson S. Burden and consequences of child maltreatment in high-income countries. Lancet. (2009) 373:68–81. 10.1016/S0140-6736(08)61706-7 19056114

[11] Hardcastle K Bellis MA Ford K Hughes K Garner J Ramos Rodriguez G. Measuring the relationships between adverse childhood experiences and educational and employment success in England and Wales: findings from a retrospective study. Public Health. (2018) 165:106–16. 10.1016/j.puhe.2018.09.014 30388488

[12] Kinge JM Modalsli JH Øverland S Gjessing HK Tollånes MC Knudsen AK. Association of household income with life expectancy and cause-specific mortality in Norway, 2005-2015. JAMA. (2019) 321:1916–25. 10.1001/jama.2019.4329 31083722 PMC6515574

[13] Mackenbach JP Kunst AE Cavelaars AE Groenhof F Geurts JJ. Socioeconomic inequalities in morbidity and mortality in western Europe. The EU Working Group on Socioeconomic Inequalities in Health. Lancet. (1997) 349:1655–9. 10.1016/S0140-6736(96)07226-1 9186383

[14] Agdertall AR. (County Statistics). Aust- og Vest Agder: Aust-Agder og Vest-Agder Fylkeskommune (Aust-Agder and Vest-Agder County). Kristiansand (2018).

[15] Saunders BE Adams ZW. Epidemiology of traumatic experiences in childhood. Child Adolesc Psychiatr Clin N Am. (2014) 23:167–84. 10.1016/j.chc.2013.12.003 24656575 PMC3983688

[16] Vederhus J-K Timko C Haugland SH. Adverse childhood experiences and impact on quality of life in adulthood: development and validation of a short difficult childhood questionnaire in a large population-based health survey. Qual Life Res. (2021) 30:1769–78. 10.1007/s11136-021-02761-0 33534031 PMC8178145

[17] Brown DW Anda RF Tiemeier H Felitti VJ Edwards VJ Croft JB. Adverse childhood experiences and the risk of premature mortality. Am J Prev Med. (2009) 37:389–96. 10.1016/j.amepre.2009.06.021 19840693

[18] Tomasdottir MO Sigurdsson JA Petursson H Kirkengen AL Krokstad S McEwen B. Self reported childhood difficulties, adult multimorbidity and allostatic load. A cross-sectional analysis of the Norwegian HUNT study. PLoS ONE. (2015) 10:e0130591. 10.1371/journal.pone.0130591 26086816 PMC4472345

[19] Roth K Harkins D Eng L. Parental conflict during divorce as an indicator of adjustment and future relationships: a retrospective sibling study. J Divorce Remarriage. (2014) 55:117–38. 10.1080/10502556.2013.871951

[20] Auersperg F Vlasak T Ponocny I Barth A. Long-term effects of parental divorce on mental health - a meta-analysis. J Psychiatr Res. (2019) 119:107–15. 10.1016/j.jpsychires.2019.09.011 31622869

[21] Walsh D McCartney G Smith M Armour G. Relationship between childhood socioeconomic position and adverse childhood experiences (ACEs): a systematic review. J Epidemiol Community Health. (2019) 73:1087–93. 10.1136/jech-2019-212738 31563897 PMC6872440

[22] Houtepen LC Heron J Suderman MJ Fraser A Chittleborough CR Howe LD. Associations of adverse childhood experiences with educational attainment and adolescent health and the role of family and socioeconomic factors: a prospective cohort study in the UK. PLoS Med. (2020) 17:e1003031 10.1371/journal.pmed.1003031 32119668 PMC7051040

[23] Hardt J Rutter M. Validity of adult retrospective reports of adverse childhood experiences: review of the evidence. J Child Psychol Psychiatry. (2004) 45:260–73. 10.1111/j.1469-7610.2004.00218.x 14982240

[24] Hardt J Vellaisamy P Schoon I. Sequelae of prospective versus retrospective reports of adverse childhood experiences. Psychol Rep. (2010) 107:425–40. 10.2466/02.04.09.10.16.21.PR0.107.5.425-440 21117468

[25] Shonkoff JP Slopen N Williams DR. Early childhood adversity, toxic stress, and the impacts of racism on the foundations of health. Ann Rev Public Health. (2021) 42:115–34. 10.1146/annurev-publhealth-090419-101940 33497247

